# DOTcvpSB, a software toolbox for dynamic optimization in systems biology

**DOI:** 10.1186/1471-2105-10-199

**Published:** 2009-06-29

**Authors:** Tomáš Hirmajer, Eva Balsa-Canto, Julio R Banga

**Affiliations:** 1 Instituto de Investigaciones Marinas, IIM-CSIC, Spanish Council for Scientific Research, C/Eduardo Cabello 6, 36208 Vigo, Spain; 2 Institute of Neuroimmunology, Slovak Academy of Sciences, Dúbravská 9, 845 10 Bratislava, Slovak Republic

## Abstract

**Background:**

Mathematical optimization aims to make a system or design as effective or functional as possible, computing the quality of the different alternatives using a mathematical model. Most models in systems biology have a dynamic nature, usually described by sets of differential equations. Dynamic optimization addresses this class of systems, seeking the computation of the optimal time-varying conditions (control variables) to minimize or maximize a certain performance index. Dynamic optimization can solve many important problems in systems biology, including optimal control for obtaining a desired biological performance, the analysis of network designs and computer aided design of biological units.

**Results:**

Here, we present a software toolbox, DOTcvpSB, which uses a rich ensemble of state-of-the-art numerical methods for solving continuous and mixed-integer dynamic optimization (MIDO) problems. The toolbox has been written in MATLAB and provides an easy and user friendly environment, including a graphical user interface, while ensuring a good numerical performance. Problems are easily stated thanks to the compact input definition. The toolbox also offers the possibility of importing SBML models, thus enabling it as a powerful optimization companion to modelling packages in systems biology. It serves as a means of handling generic black-box models as well.

**Conclusion:**

Here we illustrate the capabilities and performance of DOTcvpSB by solving several challenging optimization problems related with bioreactor optimization, optimal drug infusion to a patient and the minimization of intracellular oscillations. The results illustrate how the suite of solvers available allows the efficient solution of a wide class of dynamic optimization problems, including challenging multimodal ones. The toolbox is freely available for academic use.

## Background

Optimization plays a key role in computational biology and bioinformatics [1,2]. Dynamic optimization, also known as open-loop optimal control, seeks the maximization or minimization of a suitable performance index (which characterizes the solution quality) of a dynamic system taking into account possible equality or inequality constraints. The solution is represented by the optimal decision variables, which can be continuous (real numbers), discrete (integer numbers), or both. Continuous variables can be used to encode time-varying stimuli, while discrete variables usually represent events (like an on/off switch) or configurations. An overview of optimization in the context of computational systems biology was given by [3] and more recently by [4], the latter highlighting the need of robust and efficient dynamic optimization methods. Examples of relevant problems covered there include optimal control for modification of self-organized dynamics, optimal experimental design, dynamic flux balance analysis, the discovery of biological network design strategies and computational design of integrated biological circuits (synthetic biology).

A popular numerical approach for solving dynamic optimization problems is the control vector parameterization (CVP) method [5], which transforms the original problem into an outer non-linear programming (NLP) or mixed-integer non-linear programming (MINLP) problem, with an inner initial value problem (IVP). Solving the outer problem requires a suitable (MI)NLP solver. Since most biological systems are non-linear, the resulting optimization problems are frequently multimodal and very challenging to solve, so it is necessary to use proper global optimization methods [6].

This work presents DOTcvpSB, a user friendly MATLAB dynamic optimization toolbox based on the CVP method, which provides an easy to use environment while ensuring a good numerical performance. Users only need to define their dynamic optimization problems via a simple and compact input file which is close to the standard mathematical notation. Advanced users can tweak many configuration options for the different solvers in order to fine-tune the solution process. Although other existing toolboxes and software packages allow the definition and solution of optimization problems in systems biology (e.g. COPASI [7], PottersWheel [8] or SBtoolbox2 [9], to name a few), they are restricted to problems where the decision variables are static (time-independent). DOTcvpSB allows the definition and solution of dynamic optimization problems where decision variables are time-dependent, thus reaching a much broader class of optimization problems.

## Implementation

In this section, we first describe the class of problems considered and the framework chosen for its numerical solution. Next, we describe the organization and capabilities of the toolbox, highlighting its key features and modules.

### Mixed-integer Optimal Control Problem

The mixed-integer optimal control problem, also called mixed-integer dynamic optimization (MIDO) problem, considers the computation of time dependent operating conditions (controls), discrete – binary or integer- decisions and time-independent parameters so as to minimize (or maximize) a performance index (or cost function) while keeping a set of constraints coming from safety and/or quality demands and environmental regulations. Mathematically this is formulated as follows:

Find **u**(*t*), **i**(*t*), **p **and *t*_*f *_so as to minimize (or maximize):

(1)

subject to:

(2)

(3)

(4)

(5)

(6)

where  is the vector of state variables,  is the vector of real valued control variables,  is the vector of integer control variables,  is the vector of time-independent parameters, *t*_*f *_is the final time of the process, *m*_*e*_, *m*_*i *_represent the number of equality and inequality constraints, respectively and **g **collects all state constraints, pathway, pointwise and final time constraints and **u**_*L*_, **i**_*L*_, **p**_*L*_, **u**_*U*_, **i**_*U*_, **p**_*U *_correspond to the lower and upper bounds for the control variables and the time-independent parameters.

### Control Vector Parameterization

DOTcvpSB is based on the control vector parameterization (CVP) framework to solve the class of problems stated above. The CVP methodology proceeds dividing the control variables (**u**(*t*) and **i**(*t*)) into a number of elements and then approximating each element by means of different basis functions, usually low order polynomials. In this way the control variables are parameterized using **w**_*u *_∈ R^*ρ *^and **w**_*i *_∈ Z^*ρ*^, which become decision variables. This parameterization transforms the original infinite dimensional problem into a finite dimension (mixed-integer) non-linear programming problem that may be solved by a suitable (MI)NLP solver. Note that the evaluation of the objective function and constraints requires the solution of the system dynamics by solving an inner initial value problem (IVP).

If the outer (MI)NLP problem is convex, deterministic (gradient-based) local methods are the best alternative to efficiently solve it. In this regard, (mixed-integer) sequential quadratic programming methods, such as MISQP [10], can be considered the state-of-the-art. Nevertheless, in presence of non-convexities, local methods usually present convergence to local minima, thus requiring the use of global optimization methods.

Global optimization methods can be roughly classified in two major groups: deterministic and stochastic methods. Certain deterministic global methods can guarantee global optimality for particular classes of problems, although the computational cost becomes infeasible for problems of realistic size. They have been recently applied for the solution of MIDO problems [11,12]. Regarding stochastic methods, several works, as reviewed by [6], have illustrated their potential for dynamic optimization (DO) and, more recently, for mixed-integer dynamic optimization (MIDO) [13]. Stochastic methods usually locate the vicinity of global solutions with reasonable efficiency, but the cost to pay is that global optimality can not be guaranteed. Alternatives such as global-local hybrid methods have been presented both for DO [14] and MIDO [15], significantly improving the computational efficiency. Thus, we could summarize the current state-of-the-art in this domain by concluding that there is no silver bullet for global optimization of arbitrary MIDO problems. And this is why DOTcvpSB includes a suite of optimization solvers, following a "Swiss Army knife" approach.

Many of these optimization methods require the computation of the gradient of the objective and/or constraints with respect to the decision variables. Vassiliadis [5] proposed the use of first order parametric sensitivities to compute such information. The sensitivity equations result from a chain rule differentiation applied to the system defined in Eqns. 2 with respect to the decision variables and may be solved in combination with the original system. For this purpose, the use of Backward Differentiation Formulas (BDF) methods is very attractive since they are able to exploit the fact that the original system and the sensitivities share the same Jacobian.

### Toolbox description

**Figure 1:**
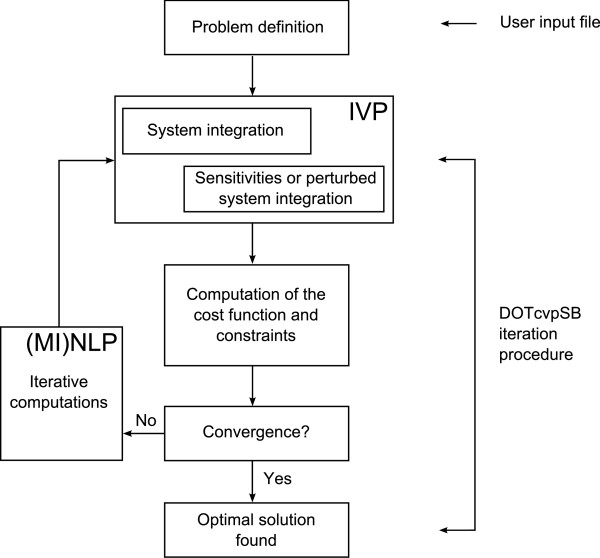
**General scheme for the solution of a DO problem in DOTcvpSB**. DOTcvpSB: solution scheme based on CVP for mixed-integer dynamic optimization problems.

DOTcvpSB has been implemented in MATLAB  following the scheme presented in Figure 1. The original dynamic optimization or mixed-integer dynamic optimization problem is solved numerically by the use of a suitable optimizer (outer iteration) which requires the solution of an IVP (inner iteration) which will in general consist on a set of ODEs plus a set of sensitivities to compute gradient information. The solution of the inner IVP is accomplished by calls to tailored solvers from the SUite of Nonlinear and DIfferential/ALgebraic equation Solvers (SUNDIALS) [16], more specifically CVODES. Since these simulations are the most computationally demanding task in the CVP method, our toolbox can automatically create compiled dynamically linked subroutines (known as MEX files in MATLAB) for the ODEs, Jacobian, and sensitivities, thus ensuring high performance.

### Key Features

The core capabilities of the toolbox can be summarized as follows:

• handling of a wide class of dynamic optimization problems, including constrained, unconstrained, fixed, and free terminal time problems described by ordinary differential equations (ODEs), as well as continuous and mixed integer decision variables;

• the inner initial value problem (IVP) is solved using the state-of-the-art methods available in SUNDIALS [16];

• the outer (MI)NLP problem can be solved using a number of advanced solvers, including *local deterministic methods*, *stochastic global optimization methods*, and *hybrid metaheuristics*;

• in addition to the traditional single optimization approach, the toolbox also offers more sophisticated strategies, like multistart, sucessive re-optimization [17], and hybrid strategies [14];

• a graphical user interface (GUI) which makes the definition and edition of a problem more easy and clear;

• possibility of importing SBML models [18];

• many output options for the results, including detailed figures.

### Description of main modules

The toolbox contains a number of modules (implemented as MATLAB functions) which can be grouped in two categories:

• utility modules: graphical user interface (GUI), simulation, and SBML-import modules;

• optimization modules: offering several optimization strategies, namely single optimization, multi-start, successive re-optimization, and hybrid optimization modules.

#### Utility modules

The utility modules offer several facilities for the definition, checking, and handling of problems. The toolbox can be operated through two equivalent approaches: by the use of the GUI, or directly from the command line (from where scripts with problem definitions can be created and executed). It also offers a module to import dynamic models from SBML files, and the imported models can be checked by a simulation module.

• **Graphical User Interface (GUI) module**: this module was developed in order to help users in the definition and execution of problems. With the help of this module, which follows an intuitive wizard-like approach, problem definitions and modifications are guided in an easy and convenient stepwise manner, especially indicated for entry users.

• **Simulation module**: this module carries out the dynamic simulation of the user-defined dynamics (plus assigned initial conditions and controls) generating the corresponding state trajectories. This module is especially useful for checking the model correctness during the definition phase, which is particularly error-prone. Typical errors like those related with units inconsistencies can be readily identified with this procedure.

• **SBML to DOTcvpSB module**: this module allows DOTcvpSB to import the systems dynamics from SBML (Systems Biology Markup Language) models [18,19]. Once a dynamic model is imported, it is necessary to check the model correctness by simulation (previous module). If everything works correctly, the user can proceed with the definition of the other terms of the dynamic optimization problem (performance index, constraints) and, finally, with its numerical solution.

#### Optimization modules

The optimization modules offers a suite of four different optimization strategies, each one with different options for the optimization solvers, following the "Swiss Army knife" approach mentioned previously. All these modules are described in more detail below.

• **Single optimization module**: this module makes a single call to one of the optimization solvers, which can be either a local deterministic or global stochastic method (see available solvers below). This procedure can be sufficient for well conditioned, convex problems, or non-convex problems which are cheap to evaluate. In any case, it is recommended as the first strategy to try with any new problem.

• **Multi-start optimization module**: this modules runs a selected optimization solver (typically a local one) repeatedly. The set of solutions (performance index values) obtained can then be analyzed (e.g. plotting a histogram) in order to check the multimodality of the problem.

• **Sucessive re-optimization module**: Sucessive re-optimization can be used to speed up the convergence for problems where a high discretization level is desired (e.g. those where the control profiles behave wildly). This procedure runs several successive single optimizations automatically increasing the control discretization, NLP, and IVP tolerances after each run.

• **Hybrid optimization module**: Hybrid optimization is characterized by the combination of a stochastic global method plus a deterministic local method. This procedures ensures a compromise between the robustness of global methods and the efficiency of local ones. This module is especially indicated for difficult multimodal problems. In any case, tweaking the hybrid method requires a deep knowledge of the solvers, and this approach will be almost always more costly (in CPU time) than the single optimization procedures using local methods (the price to pay for the increased robustness).

### Numerical optimization methods (NLP and MINLP solvers)

The toolbox provides interfaces to several optimization state-of-the-art solvers:

• local deterministic

1. IPOPT [20] implements a primal-dual interior point method, and uses line searches based on Filter methods;

2. FMINCON [21] is a part of the MATLAB optimization toolbox which uses sequential quadratic programming (SQP);

3. MISQP [10] solves mixed-integer non-linear programming problems by a modified sequential quadratic programming method;

• stochastic global

1. DE [22] uses population based approach for minimizing the performance index;

2. SRES [23] uses an evolution strategy combined with an approach to balance objective and penalty functions;

• and hybrid metaheuristics

1. ACOmi [15] is inspired by ants foraging behavior, using MISQP for local searches;

2. MITS [13] is based on extensions of the Tabu Search metaheuristic, using MISQP for local searches;

where the deterministic MISQP solver and all hybrid solvers are able to handle mixed-integer problems directly. Users can change solvers by simply changing an option in the input data structure, thus requiring no problem reformulation.

### Numerical simulation method (IVP solvers)

Forward integration of the ODE, Jacobian, and sensitivities (when needed) is ensured by CVODES, a part of SUNDIALS [16], which is also able to perform simultaneous or staggered sensitivity analysis. The IVP problem can be solved with the Newton or Functional iteration module and with the Adams or BDF linear multistep method (LMM). The Adams method is recommended for solving of the non-stiff problems while BDF is recommended for solving of the stiff problems. Note that the sensitivity equations are provided analytically and the error control strategy for the sensitivity variables could be enabled.

### Recommended operating procedure

It should be noted that, for a general MIDO formulation, there is no a priori way to distinguish if the resulting MINLP will be convex or not inside the search space considered, so the user has no clue on which optimization strategy should be using. Thus, we recommend that, for any new problem, the user follows this protocol:

• Step 1: try solving the problem with the single optimization strategy and a local deterministic method, such as FMINCON or IPOPT for DO problems, or MISQP for MIDO problems, using a rather crude control discretization (e.g. 10 elements). After obtaining a solution, repeat changing the initial guess for the control variable. If a rather different solution is obtained, suspect multimodality and go to step 2 below. If not, solve the problem again using a finer discretization. For faster and more satisfactory results regarding control discretization, use the successive re-optimization module.

• Step 2: solve the problem using the multi-start optimization module. In general 100 runs is a sensible number for this task, but for costly problems the user might want to reduce this. Plotting an histogram of the resulting set of solutions will give a good view of the problem multimodality. For clearly multimodal problems, go to step 3. If not, stop, or go back to step 1 if e.g. more refined control levels are desired.

• Step 3: use the single optimization strategy as in step 1, but use a global stochastic method, like DE or SRES for DO problems, or ACOmi or MITS for MIDO problems. If satisfactory results are obtained in reasonable computation times, stop. If the computational cost is excessive, go to step 4.

• Step 4: use a hybrid global-local strategy. More advanced users can tweak the different options to increase efficiency and/or robustness.

This protocol is especially recommended for novel users who are not familiar with numerical optimization methods. Advanced users can tweak the hybrid strategy options, or even create their own strategies combining calls to the different solvers in a MATLAB script.

## Results and discussion

This section illustrates the usage and performance of the different modules of DOTcvpSB considering several illustrative examples.

### Importing and checking a SBML dynamic model

For illustrative purposes, a dynamic model of the cell cycle [24] was chosen and imported into the DOTcvpSB toolbox. The problem is marked as BIOMD0000000005, Tyson1991_CellCycle_6var, 1831270 can be downloaded as an '.xml' file from the Biomodels database web page: .

**Figure 2:**
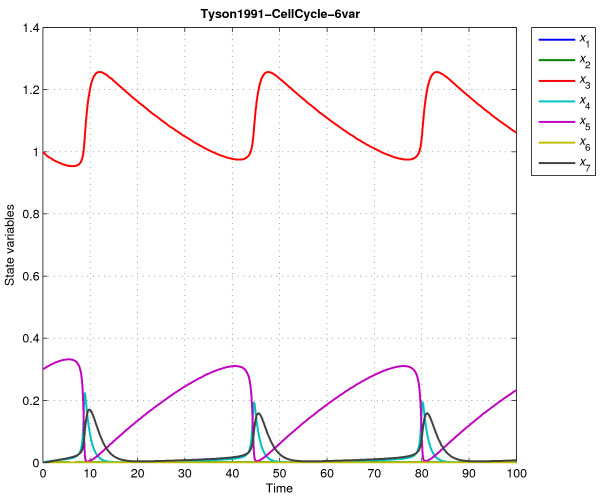
**Importing a dynamic model from SBML to DOTcvpSB**. This figure shows the dynamic behavior of the cdc2-cyclin model with 6 variables. This model was imported from a SBML file and then checked by simulation in the DOTcvpSB toolbox.

After importing it using function dotcvp_sbml2dotcvpsb, the user should perform a dynamic simulation using the simulation module to check the model. Figure 2 shows all state trajectories of the cdc2-cyclin model simulated with the constant parameters supplied in the above version.

### Single optimization

Here we solve a relatively simple problem to illustrate the usage of the single optimization strategy with a local deterministic solver.

#### Drug displacement problem

**Figure 3:**
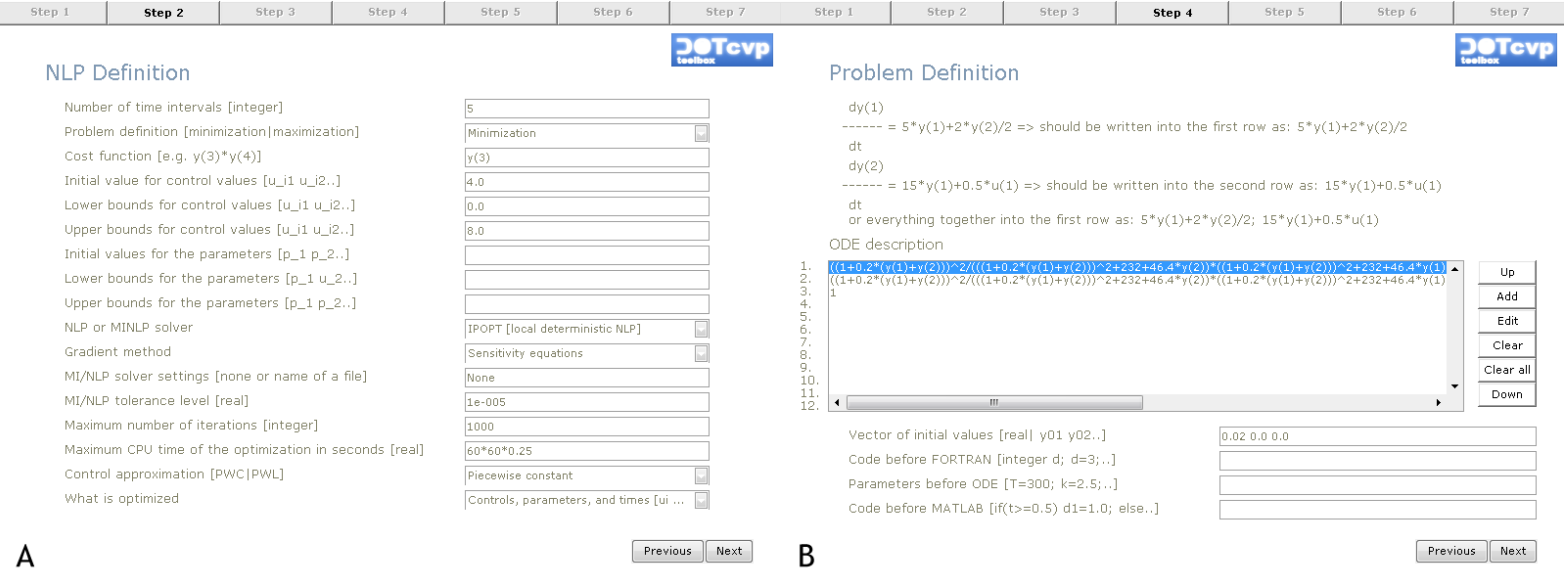
**Problem definition with the help of Graphical User Interface**. The Graphical User Interface (GUI) guides the user during problem definition or modification. The user must provide the different terms regarding the problem and the desired solution approach. Figure (A) on the left shows an screen where the optimization options are set (with NLP settings, gradient method, performance index, and bounds on the control variables and on time-independent parameters). Figure (B) illustrates how the user can introduce the systems dynamics and related options, such as initial conditions, fixed parameters.

The problem consists of finding the optimal rate of injection of a phenylbutazone infusion to minimize the time needed to reach in a patient's bloodstream a desired level of two drugs [14]. The system dynamics is described by 2 non-linear differential equations where the state variables represent the concentration of warfarin (*x*_1_) and phenylbutazone (*x*_2_). These drugs must achieve a desired value at final time, a requirement which is mathematically formulated as two end-point constraints. Table 1 shows a typical input script to solve this problem with DOTcvpSB. Alternatively, the problem can be defined (or loaded and modified) using the GUI, as presented in Figure 3. Mathematically, this is a constrained minimum time problem stated as:

**Table 1:** DOTcvpSB typical input data structure for the drug displacement problem.

% Example of the DOTcvp simple input file for the drug displacement problem		
data.name	= 'DrugDisplacement';	% name of the problem
data.odes.parameters(1)	= {'A = 232'};	% constant parameters before ODE
data.odes.parameters(2)	= {'B = 46.4'};	
data.odes.parameters(3)	= {'C = 2152.96'};	
data.odes.res(1)	= {'((1+0.2*(y(1)+y(2)))^2/(((1+0.2*(y(1)+y(2)))^2+A+B*y(2))*((1+0.2*(y(1)+y(2)))^2+A+B*y(1))-C*y(1)*y(2)))*(((1+0.2*(y(1)+y(2)))^2+A+B*y(1))*(0.02-y(1))+B*y(1)*(u(1)-2*y(2)))'};	
data.odes.res(2)	= {'((1+0.2*(y(1)+y(2)))^2/(((1+0.2*(y(1)+y(2)))^2+A+B*y(2))*((1+0.2*(y(1)+y(2)))^2+A+B*y(1))-C*y(1)*y(2)))*(((1+0.2*(y(1)+y(2)))^2+A+B*y(2))*(u(1)-2*y(2))+46.4*(0.02-y(1)))'};	
data.odes.res(3)	= {'1'};	
data.odes.ic	= [0.02 0.0 0.0];	% vector of initial conditions
data.odes.tf	= 300.0;	% final time
data.nlp.RHO	= 5;	% CVP discretization level
data.nlp.J0	= 'y(3)';	% performance index, min-max(performance index)
data.nlp.u0	= 4.0;	% initial guess for control values
data.nlp.lb	= 0.0;	% lower bounds for control values
data.nlp.ub	= 8.0;	% upper bounds for control values
data.nlp.solver	= 'IPOPT';	% ['FMINCON'|'IPOPT'|'SRES'|'DE'|'ACOMI'|'MISQP'|'MITS']
data.nlp.FreeTime	= 'on';	% ['on'|'off'] set 'on' if free time is considered
data.nlp.eq.status	= 'on';	% ['on'|'off'] switch on/off of the equality constraints
data.nlp.eq.NEC	= 2;	% number of active equality constraints
data.nlp.eq.eq(1)	= {'y(1)-0.02'};	% first equality constraint
data.nlp.eq.eq(2)	= {'y(2)-2.0'};	% second equality constraint
data.nlp.eq.time(1)	= data.nlp.RHO;	% to indicate that it is an end-point constraint
data.nlp.eq.time(2)	= data.nlp.RHO;	% to indicate that it is an end-point constraint
data.options.trajectories	= size(data.odes.res,2)-1;	% how many state trajectories will be displayed

This table shows a typical input file for DOTcvpSB. Many more options can be set, otherwise their values will be taken from defaults (defined in a file).

(7)

subject to

(8)

(9)

with  defined as follows

(10)

(11)

(12)

(13)

where the decision variable (control (u)) is constrained with lower and upper bounds set at values of 0.00 and 8.00. The desired concentrations of the drugs in the blood at final time should be equal to 0.02 and 2.00, respectively.

**Figure 4:**
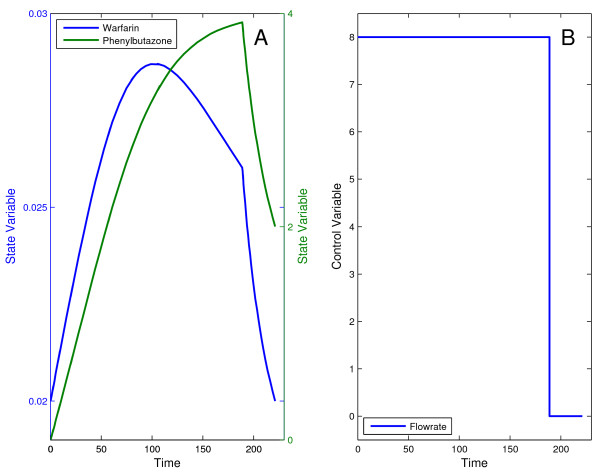
**Optimal trajectories for the drug displacement problem**. Optimal state trajectories (A) and control profile (B) for the drug displacement problem. The control trajectory infuses the desired amount of drugs into the patient's bloodstream in minimum time.

The problem was successfully solved with DOTcvpSB using the single optimization strategy with a control discretization level *ρ *= 5 and IPOPT as the NLP solver. The optimal solution found corresponds to a minimum time of 221.24 which is in good agreement with the best published result of 221.43 [6]. The optimal control profile and the corresponding state trajectories are shown in the Figure 4.

### Successive re-optimization

Here we show how to use the successive re-optimization module in order to obtain refined optimal control profiles.

#### Lee-Ramirez bioreactor

This example considers the optimal control of a fed-batch bioreactor for induced foreign protein production by recombinant bacteria. This problem was first presented by Lee et al [25], slightly modified by Tholudur et al [26], and later solved using a second order sensitivities approach [27]. The objective is to maximize the profitability of the process using the nutrient (*u*_1_) and the inducer feeding rates (*u*_2_) as control variables. Several different values for the ratio of the cost of inducer to the value of the protein production (*Q*) were published in the literature, but here we consider the particular case of *Q *= 2.5. Mathematically, the statement is to find the control trajectories that maximize the performance index at the fixed final time

(14)

subject to

(15)

(16)

(17)

(18)

(19)

(20)

(21)

where the state variables represent the reactor volume (*x*_1_), the cell density (*x*_2_), the nutrient concentration (*x*_3_), the foreign protein concentration (*x*_4_), the inducer concentration (*x*_5_), the inducer shock factor on cell growth rate (*x*_6_), and the inducer recovery factor on cell growth rate (*x*_7_). The final time is specified as 10 h. The additional constrains at the decision variables are lower and upper bounds set at the value of 0.00 and 1.00.

**Figure 5:**
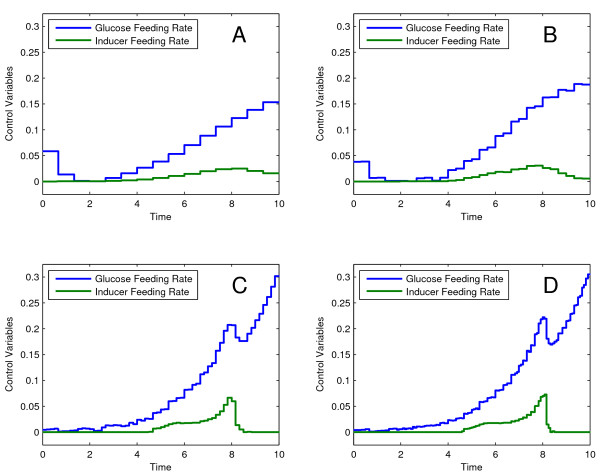
**Optimal control of the Lee-Ramirez bioreactor**. The successive re-optimization strategy was applied to the Lee-Ramirez bioreactor. Figures (A, B, C, D) show the optimal trajectories for the discretization levels 15, 30, 60, and 120, with the performance index values of 5.64058, 5.72840, 5.75707, and 5.75710.

We successfully solved this problem using the successive re-optimization strategy from DOTcvpSB and FMINCON as NLP solver, setting the initial control discretization at *ρ *= 15. The mesh increasing factor and the number of mesh refinements were set at values of 2 and 4, respectively. The results for the increasing *ρ *values are shown in Figure 5, which have the following performance index values: 5.64058, 5.72840, 5.75707, and 5.75710. These performance indexes are in very good agreement with those published in the literature.

### Hybrid optimization

Here we solve a multimodal problem using the powerful hybrid strategy, where the adequate combination of an stochastic global and a deterministic local solver allows reaching the vicinity of global solution in a reasonable computation time.

#### Drug displacement problem with path constraint

Here we consider a modified formulation of the drug displacement problem (defined above) adding an state path constraint, which is set to ensure that the warfarin concentration in the patient's blood does not exceed a dangerous level. The constraint is defined as follows

(22)

**Figure 6:**
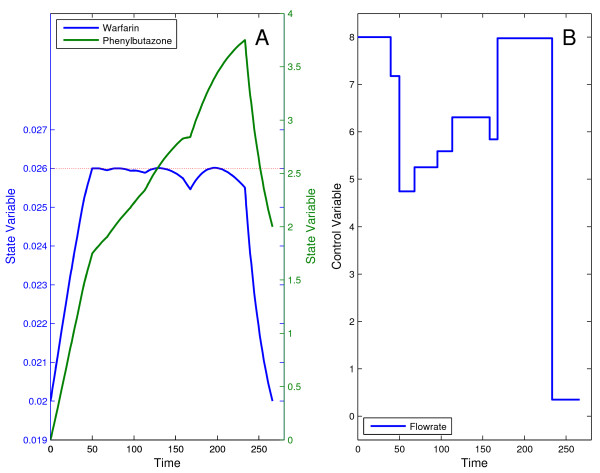
**Optimal trajectories for the drug displacement problem with path constraint**. Optimal state trajectories (A) and control profile (B) for the drug displacement problem with path constraint on the warfarin concentration amount. Solution found with the hybrid global-local strategy.

This problem has been reported to be highly multimodal, therefore its solution must be approached by the use of a suitable global method. On the other hand, a combination of a global and a local method (hybrid approach) should be more efficient. To illustrate this, we solved this problem using (i) the global DE solver (in single optimization mode) and (ii) a hybrid combining DE and MISQP solver. Using *ρ *= 10 free time intervals, both approaches converged to a similar solution, with a performance index (infusion final time) of 266.09. In addition, the inequality and all equality constraints were violated less than the pre-set tolerance of 10^-8^. But the hybrid approach was approximately 5 times faster than DE in obtaining equivalent results. It should be mentioned that these results are again in very close agreement with those presented in the above cited literature. The optimal trajectories are shown in Figure 6.

### Multistart and single optimization with a global method

The multistart strategy is a good way of checking the possible non-convexity of problems. When the multimodality of a problem has been confirmed, users can choose a global or a hybrid strategy to find a solution in the close vicinity of the global one. We illustrate all this here considering a challenging MIDO problem.

#### Phase resetting of a calcium oscillator problem: a mixed-integer dynamic optimization problem

We have considered a calcium oscillator model describing intracellular calcium spiking in hepatocytes induced by an extracellular increase in adenosine triphosphate (ATP) concentration, as originally proposed in [28] and later slightly modified and solved in [29,30]. The aim of the optimization is to minimize the intracellular oscillations behavior with the help of two binary control variables (*i*_1_, *i*_2_). The values of these variables and the time of the switching from one mode to another, together with the time-independent parameter (*p*_1_), are the decision variables. The performance index is formulated as the minimization of the state variables deviations with respect to certain desired values (see Table 2) over a fixed time horizon:

**Table 2:** Parameter values for the calcium oscillator problem

Model Parameters (Reaction Coefficients)			Weighted Coefficients	Initial Values	Desired Values
*k*_1 _= 0.09	*k*_8 _= 32.24	*K*_15 _= 0.16	*w*_1 _= 5.0	*x*_1_(0) = 0.03966	= 6.78677
*k*_2 _= 2.30066	*K*_9 _= 29.09	*k*_16 _= 4.85	*w*_2 _= 5.0	*x*_2_(0) = 1.09799	= 22.65836
*k*_3 _= 0.64	*k*_10 _= 5.0	*K*_17 _= 0.05	*w*_3 _= 15.0	*x*_3_(0) = 0.00142	= 0.38431
*K*_4 _= 0.19	*K*_11 _= 2.67	*t*_*F *_= 22.0	*w*_4 _= 25.0	*x*_4_(0) = 1.65431	= 0.28977
*k*_5 _= 4.88	*k*_12 _= 0.7		*w*_5 _= 50.0		
*k*_6 _= 1.18	*k*_13 _= 13.58		*w*_6 _= 5.0		
*k*_7 _= 2.08	*k*_14 _= 153.0				

(23)

subject to

(24)

(25)

(26)

(27)

and the time-independent parameter: 1 ≤ *p*_1 _≤ 1.3, where state variables represent the concentration of activated G-protein (*x*_1_), active phospholipase C (*x*_2_), intracellular calcium (*x*_3_), and intra-ER calcium (*x*_4_). The time-fixed parameters together with the initial concentrations, desired values of the state variables and weighted coefficients are described in detail in the Table 2. The control variables are chosen binaries (*i*_1_, *i*_2_), which refer to the concentrations of an uncompetitive inhibitor of the PMCA (plasma membrane *Ca*^2+^) ion pump and the inhibitor of PLC activation by the G-protein. The influence of the first inhibitor is modeled according to Michaelis-Menten kinetics while that of the second inhibitor is modelled with the help of the term (1 - *i*_2_), where *i*_2 _= 1 corresponds with the maximum amount of the inhibitor. An additional equality constraint was added to fix the final time at the fixed value (*t*_*F*_). The best performance index reported in [29] was 1538.00, where this reported cost function corresponds to the term . These authors reported that the system is extremely sensitive to small perturbations in the stimulus.

**Figure 7:**
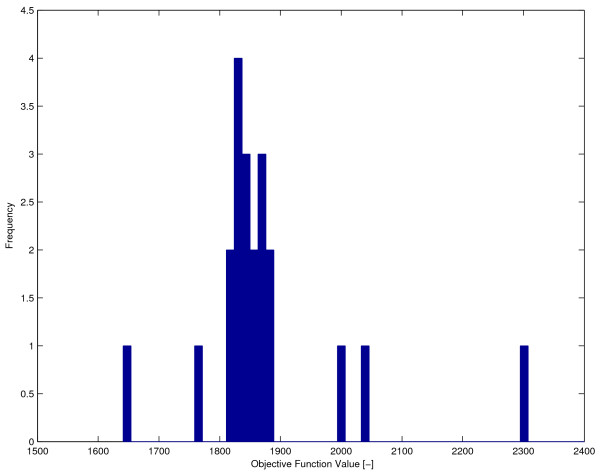
**Multistart optimization for the calcium oscillator problem**. The calcium oscillator problem was solved using the multistart module and the MISQP local solver.

We first solved this problem using the multistart module of the DOTcvpSB toolbox, using MISQP as local solver. The control discretization level was set to a value of *ρ *= 5 with free transition times and two binary decision variables for the controls. The multistart number of runs was set to 100, with randomly generated initial values for all the decision variables in each run. The set of solutions found were spread in a quite wide range, a clear sign of multimodality. The histogram of these solutions is shown in Figure 7, where performance index values worse than 2500.00 are not shown. The best value (for the reduced cost term above) obtained by the multistart was 1641.03, which is still far from the published solution reported above.

**Figure 8:**
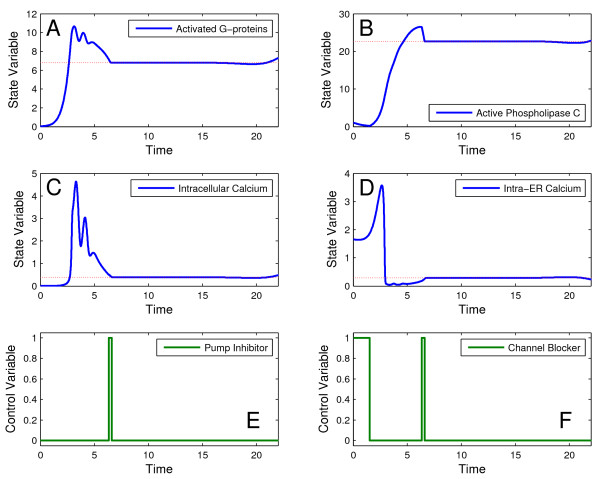
**Optimal trajectories for the calcium oscillator problem**. Optimal state trajectories (A, B, C, D) (blue lines), desired states (dotted red lines), and control profiles (E, F) (green lines) for the calcium oscillator problem with two control variables.

In a second step, we solved this problem using the MITS hybrid strategy, while keeping all the other settings as stated above. The best solution found by MITS was 1542.50, which is very close to the value reported in [29]. The corresponding optimal trajectories are shown in Figure 8 where it can be seen how the optimal control policies rapidly cancel the oscillations.

## Conclusion

Here we have presented DOTcvpSB, a MATLAB toolbox for solving dynamic optimization problems from the domain of systems biology. This toolbox is able to handle very general mixed-integer dynamic optimization formulations, thus providing the opportunity to state and solve complex problems, such as e.g. optimal control for obtaining a desired biological performance, dynamic analysis of network designs or computer aided design of biological units. Problems are easily defined via a compact input structure, or optionally using a graphical user interface.

This toolbox has been developed placing particular care in providing state-of-the-art solvers in order to ensure a good compromise between computational robustness and efficiency. DOTcvpSB offers two key and unique advantages:

• It incorporates a suite of local and global optimization solvers so as to handle a wide range of problems, including non-convex (multimodal) ones.

• It offers several optimization strategies, including single, multistart, sucessive reoptimization and hybrid methods. These strategies can be effectively used to enhance the solution of difficult multimodal problems.

The capabilities and performance of DOTcvpSB were successfully tested using several challenging benchmarks problems taken from the open literature. The results confirmed that the toolbox was able to get excellent results in reasonable computation times, showing a good compromise between robustness and efficiency.

## Availability and requirements

**Project name**: DOTcvpSB, a Software Toolbox for Dynamic Optimization in Systems Biology

**Project homepage**: The toolbox can be downloaded from the following website, which also offers documentation (installation instructions, manual, tutorial and video demos): 

**Operating system(s)**: Windows. A Linux version is planned for the near future.

**Programming language**: MATLAB versions 7.0–7.6 (2008a) is required, and the MATLAB Optimisation Toolbox and Symbolic Math Toolbox are highly recommended.

**Other requirements**: The toolbox distribution includes most of the needed external solvers: IVP solver CVODE (part of SUNDIALS suite), and (MI)NLP solvers ACOmi, DE, IPOPT, MISQP, MITS and SRES. The Optimization Toolbox is needed if the user wants to use FMINCON as a NLP solver. FORTRAN compilation to speed-up computations is secured by a combination of gnumex and MinGW, packages which are distributed with the toolbox as well. On the other hand, the Symbolic Math Toolbox is needed if automatic generation of sensitivities and Jacobian are desired (recommended). Users must install the SBML and libSMBL toolboxes in order to be able to import SBML models.

**License**: The toolbox can be obtained and used for free for academic purposes, and is under the creative commons license. The conditions of the license can be found on: 

**Any restrictions to use by non-academics**: Following the previous license.

## Abbreviations

ACOmi: Ant Colony Optimization for Mixed Integer non-linear programming problems; ATP: Adenosine TriPhosphate; BDF: Backward Differentiation Formula; CVP: Control Vector Parameterization; DE: Differential Evolution; FMINCON: Find MINimum of CONstrained non-linear multivariable function; MISQP: Mixed-Integer Sequential Quadratic Programming; GUI: Graphical User Interface; IPOPT: Interior Point OPTimizer; IVP: Initial Value Problem; LMM: Linear Multistep Method; MI: Mixed-Integer; MIDO: Mixed-Integer Dynamic Optimization; MINLP: Mixed-Integer Non-Linear Programming; MITS: Mixed-Integer Tabu Search algorithm; NLP: Non-Linear Programming; ODEs: Ordinary Differential Equations; SBML: Systems Biology Markup Language; SRES: Stochastic Ranking Evolution Strategy.

## Authors' contributions

TH wrote the source code, designed the GUI interface, and implemented the test problems. EBC and JRB conceived the algorithms, guided their implementation, and tested the toolbox. TH, EBC, and JRB wrote the manuscript and tested the final version of the software. All authors have read and approved the final manuscript.
